# Uncovering Olive Biodiversity through Analysis of Floral and Fruiting Biology and Assessment of Genetic Diversity of 120 Italian Cultivars with Minor or Marginal Diffusion

**DOI:** 10.3390/biology8030062

**Published:** 2019-08-28

**Authors:** Luca Lombardo, Gianni Fila, Nicola Lombardo, Chiara Epifani, Donald H. Duffy, Gianluca Godino, Amelia Salimonti, Samanta Zelasco

**Affiliations:** 1 Center Agriculture Food Environment (C3A), University of Trento, 38122 Trento, Italy; 2 Research and Innovation Centre, Fondazione Edmund Mach, 38010 San Michele all’Adige, Italy; 3 CREA Research Centre for Agriculture and Environment, 40128 Bologna, Italy; gianni.fila@crea.gov.it; 4 CREA Research Centre for Olive, Citrus and Tree Fruit, 87036 Rende, Italy; nicolalombardo2@libero.it (N.L.); gianluca.godino@crea.gov.it (G.G.); amelia.salimonti@crea.gov.it (A.S.); samanta.zelasco@crea.gov.it (S.Z.); 5 CREA Research Centre for Agriculture and Environment, 00184 Roma, Italy; chiara.epifani@crea.gov.it; 6 Department of Computer Science and Automation Control, University of Salamanca, 37007 Salamanca, Spain; dhduffy@usal.es; 7 Ketensis, New York, NY 10036, USA

**Keywords:** *Olea europaea* L., flowering, modelling, fruiting biology, SSR

## Abstract

The primary impetus behind this research was to provide a boost to the characterization of the Italian olive biodiversity by acquiring reliable and homogeneous data over the course of an eight-year trial on the floral and fruiting biology of 120 molecularly analyzed cultivars, most of which have either low or very low diffusion. The obtained data highlighted a considerable variability to almost all of the analyzed parameters, which given the uniformity of environment and crop management was indicative of a large genetic variability in the accessions under observation, as confirmed through the molecular analysis. Several cases of synonymy were reported for the first time, even among plants cultivated in different regions, whilst all of the varieties examined, with only one exception, showed very low percentages of self-fruit-set, indicating a need for the employment of suitable pollinator plants. Eventually, a fitted model allowed us to evaluate the clear effects of the thermal values on blossoming, particularly in the months of March and April, whereas the climatic conditions during the flowering time had only a modest effect on its duration.

## 1. Introduction

The olive tree (*Olea europaea* L.) usually blossoms rather abundantly, with up to half a million flowers per plant [1]. Nevertheless, the percentage of fruiting flowers is very low, generally below 2% [2,3,4]. This mainly comes down to genetic factors, such as pistil abortion and intraspecific self-incompatibility, which along with the summer fruit drop, biotic and abiotic stresses, and the presence of appropriate pollinators greatly influence the production potential of this andromonoecious species [5,6]. The study of floral and fruiting biology is therefore crucial from an agronomic and physiological point of view, especially considering the remarkable size of the olive germplasm, which is estimated to include 2629 accessions [7].

The scientific approach to the study of floral biology in olives traces its roots back to the first decade of the 20th century with the research conducted primarily by Petri [8] and Campbell [9], nevertheless, most of the available studies were carried out on a small number of cultivars, in vastly different environments, and over a limited number of years [10,11,12,13,14,15,16,17,18,19,20,21]. As a result, the conflicting reports regarding the classification of pollen compatibility in some cultivars, as well as the contradictory results in different areas and years [22], do not allow for definitive conclusions to be drawn. On the other hand, the few works on floral biology conducted in a comparative manner (in the same pedoclimatic conditions and cultivating techniques) [5,23,24] looked at olive cultivars with medium or wide distribution, for which many data were already available. In this sense, the present work reports on data acquired for 120 varieties that have a mostly reduced or very low local distribution—in some cases being at risk of extinction [25,26]—and which have been seldom studied, if at all. Since these data are utilized to describe, identify, and classify the cultivars, as recognized by the International Union for the Protection of New Varieties of Plants (UPOV) Guideline TG/99/4, the objective of this work is to provide a more thorough understanding of the vast Italian olive germplasm, which appears to be the richest in the world, comprising over 800 varieties [27]. Accordingly, the molecular characterization of these varieties will provide a further step forward towards the proper identification of the olive genotypes, while the knowledge of population structure and genetic variability of the olive germplasm is essential to define priorities for management and conservation of gene pools and to study the impact of domestication on olive tree genetic variability [28]. Eventually, a phenological model fitted to the reported flowering dates allowed us to characterize the cultivar-dependent variation of flowering time.

## 2. Materials and Methods

**Figure 1:**
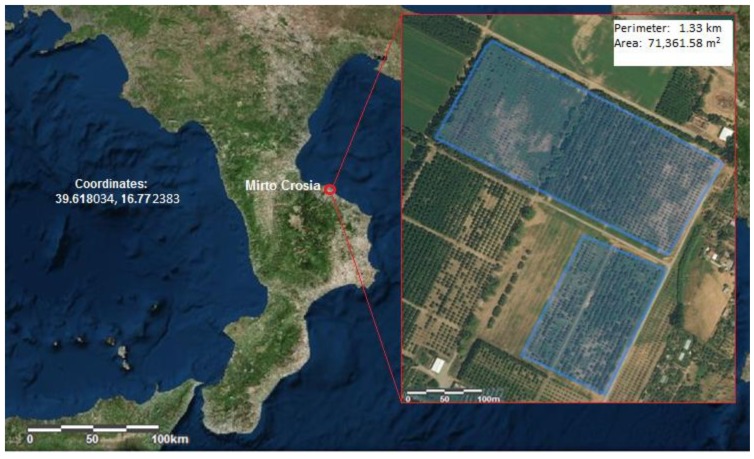
Geographical coordinates of the olive germplasm collection field.

The research was conducted within the varietal collection field of the national olive germplasm located in Mirto, on the Ionian coast of the province of Cosenza, Italy (Figure 1). The classic collection method was applied to the staging of the field: land surveying, identification of varieties and ecotypes, foundational customization, and introduction into the collection field. Moreover, materials identified by Italian scientific institutions were introduced. To date, about 500 varieties and clones have been introduced to the collection (four to five plants for each accession).

**Figure 2:**
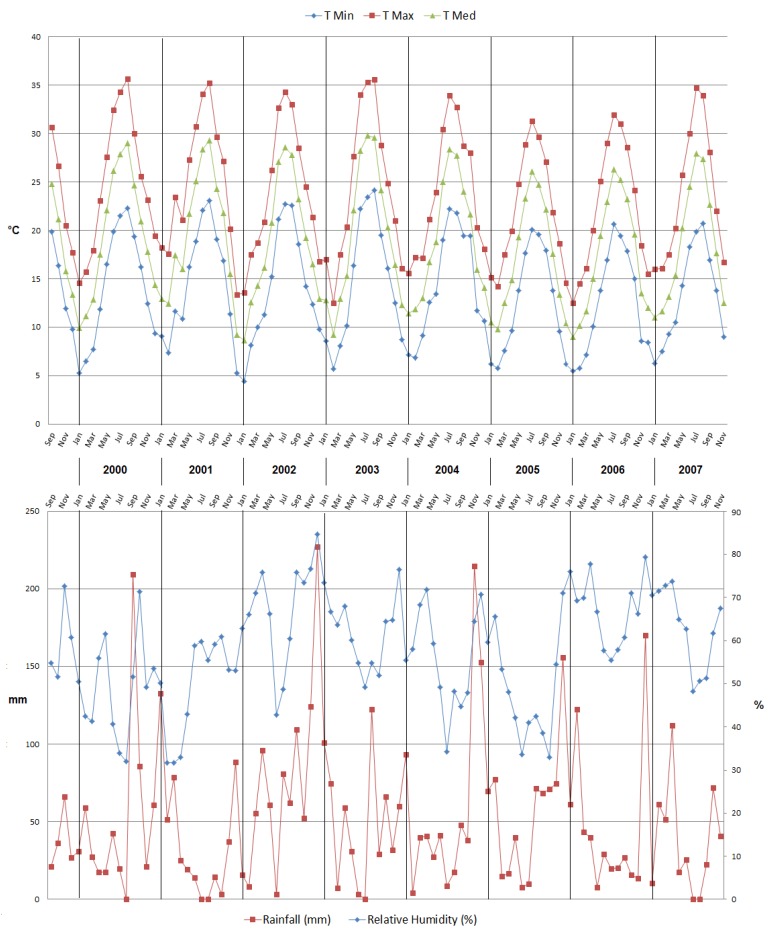
Climatic conditions during the eight-year trial.

The climate is semi-arid, with an average annual temperature of 18.9 °C and an average annual precipitation of about 500 mm, with modest year-to-year variations, which is sufficient so as to have had notable effects on some processes (the start of the blossoming, for example). Lack of precipitation, especially in summertime, was offset by a subsurface drip irrigation system. The field is maintained in accordance with sustainable management, with a vegetation cover (mowed in summertime), occasional fertilization using ternary fertilizers, and light annual pruning. The meteorological data (minimum, maximum, and average temperature, relative humidity, and precipitation amounts) for the phenological model were collected in situ by means of a meteorological station. The meteorological trend over the eight years of study is reported in Figure 2.

Measurements for each parameter were performed on five plants (four plants for rare cases of plants that are either heavily diseased or explanted) for each of the 120 cultivars selected, and were repeated for eight consecutive years, from 2000 to 2007, in order to account for any anomalous values in production and the influence of climatic conditions. The parameters considered are reported below.

### 2.1. Phenological and Physiological Characteristics

#### 2.1.1. Amount of Flowering

Visual observations were carefully conducted during the flowering phase (March and April) throughout the eight consecutive years through randomized comparisons of the differentiation percentages of the flower buds of a similar number of the plants’ tagged twigs. Specifically, every year at the time of inflorescence formation, an adequate number of twigs were tagged on the plants for a total of over 3000 inflorescences per variety. Meanwhile, an adequate number of twigs in blossom bearing an equivalent number of inflorescences were bagged in such a way as to enable the assessment of the fruit set from self-pollination.

The plants were divided into four classes: those without inflorescences (or at most with an insignificant number of inflorescences), plants with a low blossom profile (1 to 30% flower differentiation), plants with intermediate flowering (30–60% differentiation), and plants with an elevated blossom percentage (60–90% or higher).

#### 2.1.2. Timing and Duration of Flowering

The observations were carried out three times a week over the relevant period (April–June). The flower development stages were recorded according to the BBCH (Biologische Bundesanstalt, Bundessortenamt and CHemical industry)-scale [29]. In particular, the stages considered were: BBCH60: beginning of flowering—up to 10% of flowers open; BBCH65: full flowering—at least 50% of flowers open; and BBCH69: end of flowering—all petals have fallen. The data were utilized for an explorative analysis of the cultivar-dependent variability of flowering time, and to individuate date thresholds to discriminate cultivars amongst early, medium, and late flowering varieties.

#### 2.1.3. Length of the Inflorescences, Number of Flowers per Inflorescence, and Pistil Abortion

In the stage prior to flower opening, a variable number of flowering buds (depending on the extent of the individual plant’s blossom) were cut so as to have at least 200 blossoms per variety every year (which correspond to an average of over 3400 flowers for every variety) before being transported to the laboratory to tally the length of each inflorescence, the number of floral sprouts per inflorescence, as well as the pistil abortion percentage.

#### 2.1.4. Percentage of Fruit Set from Open and Self-Pollination

Fifteen to 20 days after the blossom process had concluded (in June), the number of small fruits present on the tagged twigs as calculated along with the percentages of fruit set per inflorescence and per number of fertile flowers. At the same time, the bags were removed, and as mentioned above, the small fruits on the selected twigs were counted and compared with the total number of flowers and flowering buds. Eventually, the self-pollination index (SI) was calculated by taking the percentage of small fruits obtained through self-pollination and dividing it by the percentage of those obtained through open pollination. Values nearing 1 indicate a heightened degree of self-fertilization; values equaling or approaching 0 indicate a self-sterile variety.

#### 2.1.5. Fruit Drop

The summer fruit drop was evaluated between July and September depending on the variety, observing the number of small fruits that remained on the tagged small branches during the first growth phase.

#### 2.1.6. Drupe Weight

The harvest of the drupes took place between October and December (depending on the variety) in order to have the olives at the same degree of veraison. Between 30 and 40 drupes per plant were weighed so as to have at least 150 values per variety each year.

### 2.2. Model-Based Phenological Characterization

Cultivar-dependent variation of flowering time was further characterized by means of a phenological model fitted to observational full flowering dates. In this way, the cultivar earliness classification was enriched with physiologically based information by providing quantitative estimates of the specific thermal requirements.

The model was chosen among a set of 11 candidate models of varying complexity, which are listed in Table S1 of the Supplementary Materials. All models were fitted to data and ranked according to the Akaike Information Criterion (AIC), which weighs the fit of a model with its complexity; when models of different complexity give similar results, the simplest model, i.e., the one with the lowest number of parameters, is preferred. The evaluation methodology is described in detail in the Supplementary Materials.

### 2.3. Molecular Characterisation via SSR(simple sequence repeat) Analysis

Sampled leaves were dried using silica gel ground to a fine powder and then stored at −80 °C until the time of analysis. Total genomic DNA was extracted from leaves using a commercial kit (Plant DNA Mini Kit, Qiagen, Germany). The DNA quality was checked with a NanoDrop 2000 spectrophotometer (Thermo scientific, Waltham, MA, USA). A set of twelve labelled microsatellites (SSRs), most of them widely used in literature, were chosen based on their amplification consistency via polymerase chain reaction (PCR), polymorphism, and ease of allele scoring in both conventional and multiplex amplification strategies [30,31,32,33,34,35]: DCA3- 6Fam, DCA5-VIC, DCA8-VIC, DCA9-6Fam, DCA11-PET, DCA16-VIC, DCA18-6Fam [36], GAPU71B-6Fam, [37], UDO12-NED, UDO15-NED [38], EMO090-6Fam [39], and OLEST23-PET [40] loci were used in this work. Different combinations of three SSR loci were used in a multiplex PCR amplification strategy, except for DCA9-6Fam and DCA16-VIC, which showed allele drop-out during multiplex amplification. Multiplexed PCRs were carried out in 15 μL final volume using a thermal cycler (GeneAmp PCR System 9700 Applied Biosystems Inc., Foster City, CA, USA). The reaction mixture was composed of 10 ng of template DNA, 10X PCR buffer, 2 mM MgCl2, 2.5 mM dNTPs, 10 μM of forward and reverse primers, and 5U/µl Taq polymerase. The PCR thermal profile was programmed as follows: a first step at 94 °C for 5 min, 30 cycles at 94 °C for 30 s, 55 °C for 30 s, and 72 °C for 40 s. The last step included 7 min of incubation at 72 °C.

Two reference varieties (Leccino and Frantoio) were included in PCR amplification to check experimental conditions (data not shown). The GeneScan 500 LIZ (Life Technologies, Carlsbad, CA, USA) was used as internal standard, and amplification products were separated on an ABI PRISM Genetic Analyzer 3130xl (Applied Biosystems Inc., Foster City, CA, USA). The allelic assignment was performed using GeneMapper 3.7v software. Standardization of raw data was conducted in comparison to the authenticated molecular profiles of Leccino and Frantoio reference varieties in accordance with Ben Mohamed et al. [33].

#### Molecular Data Analysis

Genetic diversity was evaluated using a cluster analysis of the 120 SSR profiles scored. A similarity matrix using Dice’s coefficient [41,42] was first obtained and used to determine the cluster analysis based on the unweighted pair group method with arithmetic mean (UPGMA). A dendrogram and cophenetic correlations were obtained using PAST software v.2.12. The number of alleles detected per locus (Na), the observed (Ho) and expected (He) heterozigosity, polymorphism information content (PIC), the number of null alleles (F null), and the deviation from the Hardy–Weinberg equilibrium (HW) corrected using the Bonferroni method were determined using Cervus v.3.0.7 [43,44,45].

The Wright’s inbreeding coefficients, Fis, Fit, and Fst, and gene flow (Nm) estimates were calculated using PopGene 1.32 [46].

A population structure analysis was also conducted using STRUCTURE v. 2.3.4 software [47] to establish the Bayesian relationships amongst the 120 Italian varieties. The admixture model with correlated allele frequency and a burn-in length of 100,000 followed by 100,000 runs at each K with three iterations for every K were used, with K ranging from 1 to 12. The true value of K was determined using Structure Harvester web version 0.6.93 [48].

Eventually, a parentage analysis was computed using Cervus v.3.0.7 software. An approach based on the LOD (logarithm of the odds) score significance was adopted and the following parameters were run: (i) number of offspring: 100,000; (ii) number of candidate parents: 120; (iii) proportion of candidate parents sampled: 0.4; (iv) proportion of loci typed: 0.7208. Default values were adopted for the parameters “proportion of loci mistyped” and “error rate in likelihood calculations”. The relaxed and strict confidence levels were set to 95% and 99%, respectively.

## 3. Results

### 3.1. Phenological and Physiological Characteristics

Table 1 shows the average values of the observed characters related to the floral biology of each of the 120 cultivars, along with the indication of the region of origin of the material used for the propagation or the alleged origin of the accession. The research results reveal that all of the examined features showed a wide range of variability. The most significant findings for the individual parameters are indicated below.

#### 3.1.1. Amount of Flowering

The amount of flowering was high in more than half of accessions, with variable percentage of flower differentiation ranging from 60% to over 90% of buds. The highest and most constant values were in the cultivars of Aitana, Cavalieri, I77, Nasitana a frutto grosso, Ogliastro grande, Olivone di Viterbo, Ritonnella, San Benedetto, and Tombarello.

Fifty-five varieties showed a medium amount of bloom, whilst the flowering was low in only two varieties (Cornia and Zarbo), an indication of late entry into production.

#### 3.1.2. Timing of Flowering

The duration of the interphase, i.e., the time needed to pass from the beginning to full flowering, is almost constant amongst the varieties and over the years, equaling 6–7 days (the average duration of the interphase is 6.2 days, with a very small variability).

The stable relationship amongst flowering phenophases indicates the presence of a mechanism regulating the development pacing, which was further analyzed through modeling analysis (see below).

A negative correlation (Pearson’s r = −0.84) was observed between the timing of BBCH60 (as well as BBCH65) and the average temperature in March, namely during the phase of development of the flower buds. In confirmation of this, an even higher correlation was found between the accumulated growing degrees (growing degree-days -GDD-) for the month of March and BBCH60 (r = −0.87). Growing degree-days for the month of March have been calculated with the formula:

(1) $$GDD=\overset{\mathrm{March}\;1^{st}}{\underset{\mathrm{March}\;31^{st}}{\sum }}\left[\frac{T_{max}+T_{min}}{2}\right]-Tb$$

where base temperature (Tb) = 8. This threshold temperature was chosen on the grounds of previous literature, which indicate that for Southern Italy temperature values range between 6 and 9 °C [49,50,51].

A polynomial equation can be proposed here as:

(2) $$y=0.37x^{2}-99.34x+6,717$$

with R^2^ = 0.81, where *y* is the beginning of flowering (number of the day in the year) and *x* is the GGD for the month of March. A rather similar correlation (Pearson’s r = −0.75) was observed between BBCH60 and rainfall in March. This may suggest that in the flowering-inducing hot period, given the same temperature, a greater amount of rainfall favors an earlier opening of the flowers as a likely effect of factors such as an increased absorption of nutrients from the roots.

Once it was established that the annual data were normally distributed, we established the lower and upper limits in order to divide the varieties into “early” (E), “medium” (M), and “late” (L) for each year. With this criterion, each variety is identified by a string—a series of eight combinations (one for each year) of characters E, M, and L. The threshold value was identified according to two parameters: (i) the probability (α) associated with the event was sufficiently low; (ii) each variety presented a maximum of two characters simultaneously (M + L or M + E) during the eight years. In our case, the optimal probability value corresponded to α = 9% and the fixed limits were:

(3) $$lim_{sup}=m+1.28*s\;and\;lim_{inf}=m-1.28*s$$

where *m* is the mean and *s* is standard deviation. The Bernoulli distribution was further employed as a reasonable criterion for unambiguous classification of M + L or M + E varieties, where it provides the probability that an event having an a priori probability of occurring in a test may occur x times in N repeated tests by pure effect of the case. Based on these considerations, we could reasonably classify the following varieties.

Early varieties: Abunara, Caprina di Casalanguida, Dolce di Andria, Giusta, Provenzale, Rosciola Coltodino, Riminino, Sammartinenga, San Benedetto, Spezzanese, Tonda dolce, and Paesana Bianca;

Late varieties: Ascolana dura, Aurina, Caprina Vastese, Carpellese, Erbano, Fosco, Gnagnaro, Grossa di Venafro, Morellona di Grecia, Palmarola, Resciola di Venafro, Saligna, and Santa Maria.

**Figure 3:**
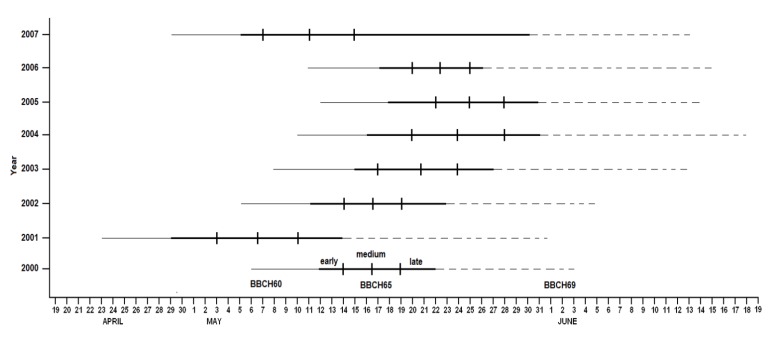
Phenogram illustration of flowering timing and duration over the eight years, with the indication of the limits calculated for early, medium, and late varieties.

The flowering phenogram (Figure 3) highlights the significant differences found through the years as a result of the climatic trend. This trend has been further investigated through a fitted phenological model.

#### 3.1.3. Duration of Flowering

The same methodology used to set the optimal threshold for the identification of early and late flowering varieties was implemented to investigate the overall duration of flowering (BBCH60-BBCH69 interphase) and whether some olive varieties have intrinsic characteristics of “short flowering” or “long flowering”. Again, a 9% probability threshold allowed us to distinguish 15 varieties with a short duration of flowering, including Aurina, Gnagnaro, Morellona di Grecia, Procanica and Rizzitella. Eighteen cultivars had a longer flowering period, amongst them Abunara, Dolce di Andria, Gentile dell’Aquila, Rotondella campana, and Tunnulidda, while 87 showed medium flowering. The varieties of Resciola di Venafro Aurina, Caprina Vastese, Fosco, Gnagnaro, Morellona di Grecia, Resciola di Venafro, and Santa Maria presented both late and the short flowering features, whilst the varieties Dolce di Andria, Provenzale, Sammartinenga, San Benedetto, and Spezzanese had early and long flowering. In general, data analysis relating to timing and duration of flowering highlighted a certain trend towards long flowering for the cultivars with early anthesis and typically short ones for late-blooming varieties.

#### 3.1.4. Length of Inflorescences and Average Number of Flowers per Inflorescence

The length of the inflorescences was on average 29.6 mm, ranging from 20 (Olivo di Castiglione) to 46.65 (Riminino) mm. Regarding the number of flowers per inflorescence, this parameter varied from 10.34 to 28.78, with a general average being equal to 17.59 ± 3.86.

Amongst the varieties with small inflorescences were Cornia, Olivo di Castiglione and Pennulara; those with a higher number of flowers were Grossa di Venafro, Riminino and Olivone di Viterbo, all with an average of over 28 flowers.

#### 3.1.5. Pistil Abortion

The percentage of pistil abortion for these 120 varieties turned out to have extreme variability, from 1% to 90%, with a general average of 38.72%.

The varieties with a lower incidence of morphological sterility included Procanica (1.07%), Crognolo (1.41%), Remugnana (2.26%), and Palmarola (2.60%). Those with a higher percentage of abortion were Oliva grossa (90.75%), Sivigliana da olio (89.24%), and Olivone di Viterbo (88.93%).

#### 3.1.6. Fruit Set by Open Pollination

The fruit set by open pollination, expressed in fruits formed per hundred inflorescences, was extremely diversified, varying from 2.27% to 115.48%, with a general average of 44.86%.

The varieties with the highest productive potential —with percentages of fruit set over 100%— were Grappolo (115.48%), Racioppa della Basilicata (107.62%), and Arnasca (103.34%), followed by Nebbia, Procanica, and Caprina Vastese, with fruit sets over 90%.

The varieties Oliva grossa, Pizzutella, Ritonnella, Oliva da mensa, Vigna della Corte, and Nasitana a frutto grosso showed the lowest fruit sets at below 15%.

If fruit set is expressed in drupes formed per 100 fertile flowers, a parameter calculated to evaluate the combined incidence of the cytological and factorial forms of sterility, then the values obtained were obviously lower, and the variability reduced, ranging from 1.04 to 16.67%.

The varieties with fruit set per fertile flower at over 10% were Pesciatino, Olivastro Frentano, Sivigliana da olio, Olivone di Viterbo, Tenacella, and Grappolo; those with fruit set below 2% were Pizzutella, Nasitana a frutto grosso, Oliva grossa, Lumiaru, Aitana, Vigna della Corte, Olivo di Castiglione, and Oliva di Casavecchia.

#### 3.1.7. Fruit Set from Self-Pollination

The percentage of self-fertility for the accessions considered in this study, intended as drupes formed per 100 bagged flowering buds, varied from 0 to 33.76.

Nine varieties displayed a total self-incompatibility, namely Ascolana dura, Caiazzana, Dolce di Cerchiara, Faresana, I77, Oliva grossa, Ruveia, Sammartinenga, and Sivigliana da olio; only 10 varieties had a fruit set scope exceeding 10%, and of these, only two surpassed 30%,namely Cellina di Rotello and Tenacella.

These data clearly indicate that all 120 varieties are associated with an adequate number of suitable pollinator varieties.

Fruit set from self-pollination, expressed as fruits formed per 100 fertile bagged flowers, varied from 0 to 4.64; only 11 varieties surpassed 1%, and the ones with higher values were Pennulara (2.38%), Olivone di Viterbo (2.49%), and Tenacella (4.64%).

#### 3.1.8. Self-Pollination Index

This index generally yielded very low results. Indeed, 22 varieties had a result equal or close to zero, 93 had a result below 0.4, and four were between 0.4 and 0.5; one exception is the Calabrian variety Pennulara, which registered a value of 1.01, indicative of a self-fertility identical to open fertilization, and therefore not in need of pollinizers.

#### 3.1.9. Fruit Drop

The summer fruit drop ranged between 0 (Caprina di Casalanguida, Caprina vastese, and Vigna della corte) and 83.69% (Biancolilla), with an average value of 32.43%, confirming this phase as a crucial step for overall production.

#### 3.1.10. Drupe Weight

This parameter varied from 0.79 (Gnagnaro) to over 6 g (Agristigna, Abunara, and Oliva grossa), with an average value of 3.13 g.

### 3.2. Model Evaluation

In terms of prediction accuracy, i.e., the capacity to predict the flowering date, all the models behaved better than the null model (Table S2 in the Supplementary Materials). The traditional GDD model, with an optimized base temperature of 2.25 °C for all cultivars, was the best performing model, as it showed the lowest Akaike Information Criterion index (AIC) [52], which weighs the fit of a model versus its complexity. Variability among cultivars could therefore be characterized through only one parameter, corresponding to the cumulated GDD from January 1 until full flowering date.

**Figure 4:**
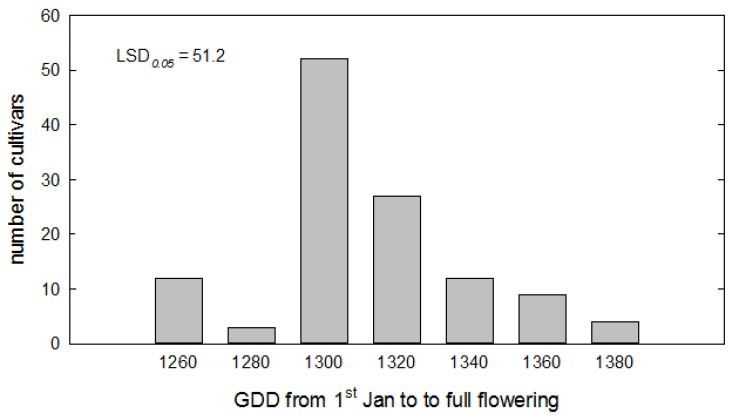
Distribution of cultivars according to the heat requirement to flower, expressed as growing degree days (Tbase = 2.25 °C) cumulated after January 1 up until full flowering.

The model was re-optimized using the whole 8-year dataset to obtain a final parameterization. The range of GDD sum, expressing the thermal requirements of the cultivars, was plotted in Figure 4, which shows the number of cultivars with specific exigencies, while the complete table with values for all cultivars is reported in Table S3 of the Supplementary Materials.

The thermal requirement varies between 1251 and 1381 °C d.

Based on the optimized thermal time, the earliest cultivar (GDD sum < 1260) were Tonda dolce, Rosciola coltodino, Giusta, Spezzanese, Dolce di Andria, San Benedetto, Sammartinenga, and Caprina di Casalanguida, while the latest ones (GDD sum > 1350) were Grappolo, Piangente, Grossa di Venafro, Saligna, Cellacchia, Santa Maria, Ascolana dura, Erbano, Racioppa, Fosco, Aurina, Gnagnaro, and Carpellese.

The thermal requirements to complete flowering (i.e., GDDs from beginning to end of flowering) were variable among the cultivars (*P* < 0.001), ranging between 188 and 316. Cultivars with the highest thermal requirements (GDDs > 300 °C d) were Cacaredda, Rustica, and Tunnulidda, whereas those with the shortest requirements (GDDs < 195°C d) were Lumiaru, Santa Maria, Aurina, Caprina vastese, and Tenacella.

No significant correlation was found between the GDD required to complete flowering and those to begin flowering.

### 3.3. SSR Diversity and Population Structure Analysis

**Figure 5:**
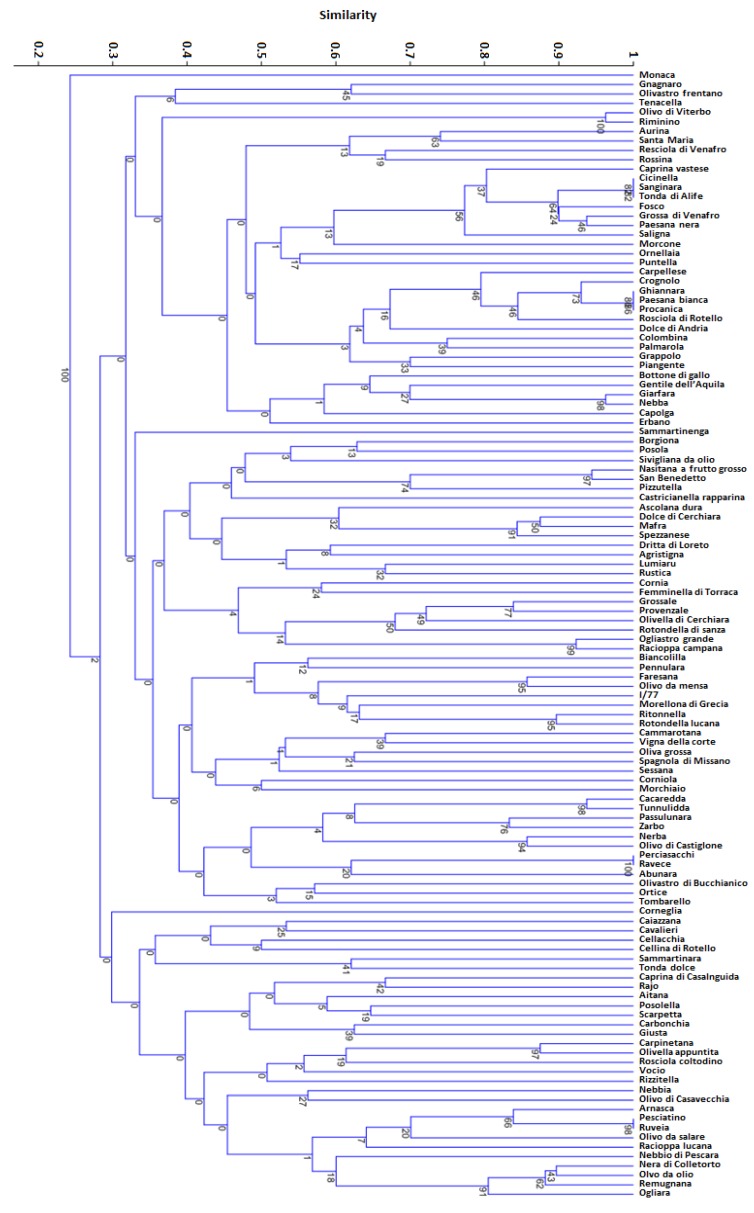
Dendrogram of relationships among the 120 olive varieties.

Molecular analysis conducted with a set of 12 microsatellites markers highlighted several cases of synonymy (Figure 5), although both the mean of the polymorphic information content (PIC = 0.73) and the mean number of alleles per locus (Na = 11.75) were relatively high (Table 2). The mean expected heterozygosity (He) was 0.77 (ranging from 0.51 for OLEST23 to 0.9090 for DCA9), and the mean observed heterozygosity (Ho) was also 0.77 (ranging from 0.4 for OLEST23 to 0.97 for DCA8). In eight cases Ho was higher than He (DCA3, DCA5, DCA8, DCA18, GAPU71b, EMO090, DCA9, UDO12), indicating high genetic variability amongst the cultivars analyzed. The probability of null alleles ranged from −0.02 to 0.3. Significant loss of heterozygosity was observed at the DCA16 and DCA11 loci; in this latter marker, the highest null allele value was found (Table 2).

The fixation indices Fis, Fit, and Fst showed a mean of −0.14, 0.01, and 0.13, respectively. The positive value of the Fst index seems to indicate a certain degree of differentiation between populations, as indicated by structure analysis results. The gene flow parameter (Nm) was on average 1.64. indicating that a gene movement occurred among populations.

Allele frequencies varied from a minimum of 0.0042 to a maximum of 0.52, with at least one rare allele for almost all the loci, aside from the locus UDO12. The most frequent allele was 206 bp at the locus DCA5. Unique alleles found and the corresponding varieties are shown in Table 3.

The dendrogram was divided in two main groups (Figure 5). The first one included the Monaca cultivar alone. The second one was divided into three subgroups with a large degree of genetic diversity. Considering a threshold of about 0.2–0.25, related to an allele difference of 1 or 2 alleles in genetic diversity [31,38], 16 putative synonymous groups (Table 4) were distinguished through cluster analysis, with a Dice similarity index ranging from 0.77 to 1.

For completeness of information, considering a wider dataset of over 300 Italian varieties whose genetic profile has been previously characterized in our laboratories [53], the cultivar Scarpetta (from Basilicata) showed the same molecular profile to Santagatese (from Sicily) likewise Carpellese (from Campania) to Correggiolo (from Emilia Romagna), Palmarola (from Basilicata) to Leccino (from Toscana), Fosco (from Lazio) to Moraiolo, Morinello (both from Tuscany) and Paesana nera (from Molise), and Paesana bianca (from Molise) to Frantoio (from Umbria).

**Figure 6:**
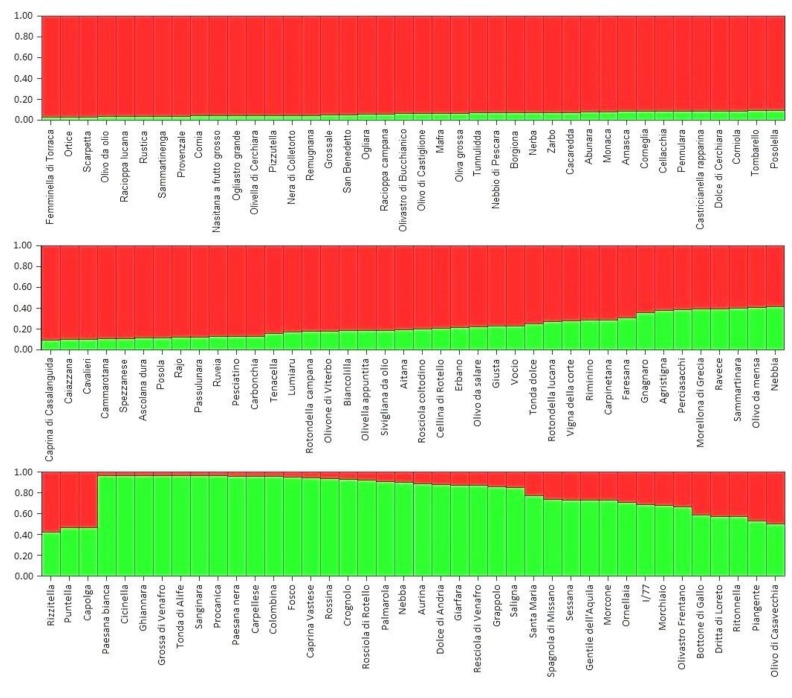
Population structure analysis showing the two differentiated groups (Red and Green) and the list of red, green, and admixed cultivars.

The population structure analysis detected two main groups (K = 2), termed “Red” and “Green”, and a few admixed accessions. Overall, this is consistent with the cluster analysis. The “Red” group included most of the cultivars from Abruzzo, Basilicata, Campania, and Sicilia, as well as all of the olive cultivars from Calabria (Figure 6). With the exception of the Calabrian accessions, no clear geographic division was highlighted by the structure analysis. However, excluding rare alleles (allele frequency 0.042–0.084), the following alleles were not found in the Green group, thus confirming a certain degree of genetic differentiation: 245bp (DCA3), 200 bp, 210bp, 212bp, 214bp (DCA5), 127bp, 131bp, 145 bp, 157 bp (DCA8), 122bp, 154bp, 166bp, 168bp, 176bp, 178bp (DCA16), 171bp, 183bp (DCA18), 186bp, 192bp, 198bp (DCA9).

**Figure 7:**
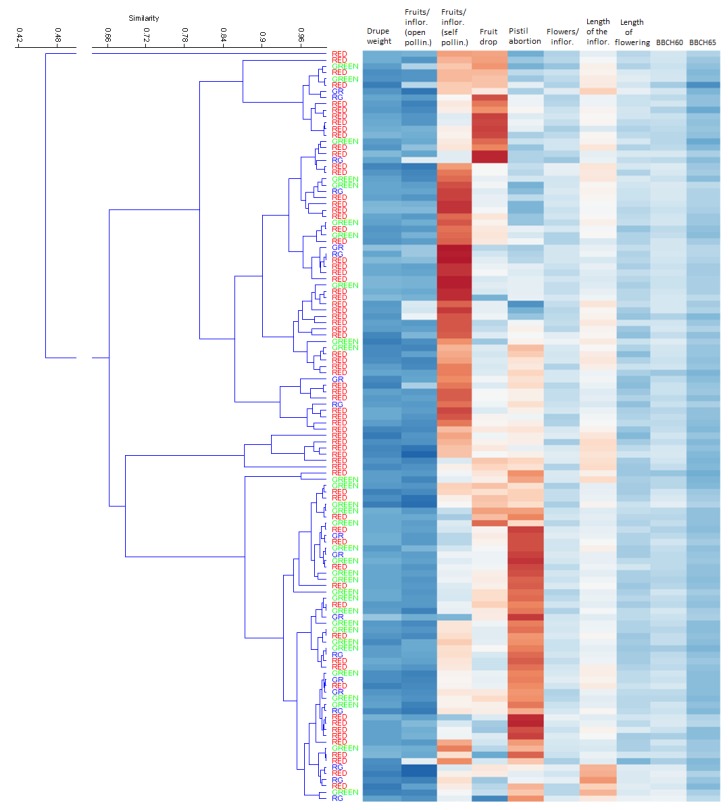
Hierarchical clustering of the principal phenological characters investigated in this work. Columns are clustered using correlation similarity and average linkage (UPGMA). GR -dominance of Green over Red- and RG -dominance of Red over Green-: admixed accessions.

A correspondence of this genetic division into two major groups has been sought for the physiological characteristics through means of hierarchical clustering (Figure 7). Pearson’s correlation coefficient was used to create a pairwise percentage similarity matrix and the dendrogram was derived using the unweighted pair group method with arithmetic averages (UPGMA). Although the distinction between these two groups was not so clear, a certain degree of separation could nonetheless be observed. Specifically, using a 0.8 similarity level, three clusters composed of six, 60, and 53 cultivars were highlighted, plus a single cultivar that did not cluster with others. The six-varieties group consisted only of “Red”, which accounted for 70% in the 60-cultivars group and 41% in the third cluster. This latter group contained the majority (68%) of the “Green” varieties, accounting for 40% of the cluster, compared with 17% within the wider group.

The non-exclusion probability between two unrelated individuals (NE-I) and two hypothetical full siblings (sibling identity, NE-SI) ranged from 0.021 (DCA9) to 0.34 (OLEST23) and from 0.31 (DCA9) to 0.6 (OLEST23), respectively. Parentage analysis revealed critical LOD scores of 18.99 and 16.43 for the parent pair analysis, with unknown sexes for strict (99%) and relaxed (95%) confidence levels. The main putative pairs of parents, with a maximum of one mismatch for each one, are shown in Table 5. The putative parents of 21 cultivars were assigned. In the case of seven cultivars, the offspring and one of the two parents shared the same region of origin, whilst in only one case did the two parents belong to confining regions.

Of note is the finding that three Sicilian cultivars, Aitana, Abunara, and Zarbo, are very likely siblings; their putative parents were Giarfara and Scarpetta from Sicily and Basilicata, respectively.

## 4. Discussions

### 4.1. Bio-Agronomic Characterization

An overall examination of all the reported data highlights that almost all of the analyzed parameters showed considerable variability, which given environmental and crop-management uniformity, is indicative of a large genetic variability in the accessions under observation.

All of the examined varieties, with one single exception (Pennulara), showed low percentages of self-fruit-set, indicating the need for the employment of suitable pollinator plants, whereas the flowering time amongst the different cultivars in the same environment was found to vary from three to four weeks (flowering scalarity).

On average, the fruit set per flowering bud was of an order 10 times greater than that per fertile flower, be they open-pollinated or self-pollinated, whilst the fruit set by open pollination was approximately 13 times greater than that from self-pollination. This confirms, from an agronomic point of view, the need for the presence of pollinators, and particularly the need for the selection of suitable pollinators, whereas some cultivars are genetically or physiologically (e.g., asynchronous flowering phases) inter-incompatible [54,55]. For this reason, it must be considered that in our trial, open pollination was favored by the presence of hundreds of different cultivars compared to a typical olive grove. Moreover, even in self-compatible varieties, open pollination might be nonetheless advantaged by the fact that ovary receptivity begins even before the opening of the flower, lasting for five to seven days, whilst the maximum emission of pollen occurs three or four days after the opening of the flowers [56].

The data on the fruit set calculated per fertile flower highlighted that regardless of the imponderable likelihood of pollen grains settling on the fertile flower during receptivity, the aspects linked to cytological and factorial sterility—already described for olive trees [57]—quite significantly affect the chances of fertilization of the ovule, and consequently (together with the aforementioned morphological sterility), the plants’ productive potential. Indeed, in addition to cases of infertility due to climatic reasons (environmental sterility) or erroneous cultivation techniques, there are those cases that are due to genetics—morphological, cytological, and factorial sterility. Morphological sterility manifests itself in both partial and total pistil abortions (gyno-sterility), and in either absent or deficient stamen and pollen development (andro-sterility). Cytological sterility depends on disturbances in meiosis during the sporogenesis processes for irregular matching of chromosomes. The flowers are morphologically normal, but the anther emits little pollen, which is not very germinable or sterile. In the case of factorial sterility, the pollen, whilst being vital and germinable, is incapable of fertilizing the flowers of the same cultivar (self-incompatibility) or of any other non-similar cultivar (inter-incompatibility).

**Figure 8:**
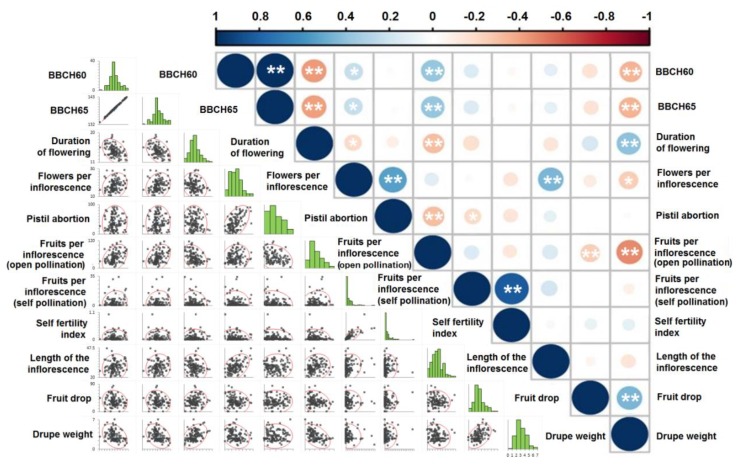
Correlation matrix based on Pearson’s product-moment correlation coefficient (r) between paired variables, across the eight-year trial. Asterisks denote statistical significance at 95% (*) and 99% (**) confidence levels.

With regards to the correlation between the number of flowers per inflorescence and pistil abortion, a positive and statistically significant correlation of 99% was found (Figure 8). This correlation is even more striking for the extreme values of the two parameters. Indeed, in the nine cultivars with flowering buds bearing an average of 24 flowers, the percentage of pistil abortion ended up being equal to 67.57%, whilst in the 10 varieties with fewer than 13 flowers per inflorescence, the average abortion percentage turned out to be barely 22.78%. On the other hand, in the 11 varieties with over 75% abortion percentage, the average number of flowers per inflorescence was equal to 23.03, whilst in the 12 varieties with pistil abortions under 10% the average number of flowers per inflorescence was 15.21. This is in contrast to what has been previously suggested by Reale et al. [58]. Eventually, drupe weight statistically significantly negatively correlated with fruit set in open pollination and time of flowering, whilst positively correlating with fruit drop and the duration of flowering. Therefore, varieties with small drupes will generally produce more fruits that will be less prone to drop.

Fitting the GDD model on the whole dataset allowed us to rank cultivars according to the thermal requirement required to reach full flowering. Overall, the GDD requirement ranged between 1090 and 1380 °C per day, but it is to be noted that two-thirds of the cultivars under study (67.5%) exhibited a much narrower variation, between 1292 and 1330 °C per day (Figure 4). As a consequence of this, most flowerings are highly concentrated over time, which could have further contributed to the high level of open pollination mentioned above, beyond the high cultivar concentration in the same grove.

Another aspect to be evidenced is that fitting the GDD model converged to base temperatures between 1 °C and 4.5 °C (not shown), with 2.25 °C as the average. In general, higher temperatures are reported, varying from 6 °C to 12.5 °C [49,59].

Calculation of base temperature may give varying results depending on the calculation methodology, and currently there is no agreement as to the best values to use [60,61,62].

Duration of flowering, expressed in thermal time, was also found to be cultivar specific, with no apparent correlation with earliness, unlike what was found when the period was expressed in calendar days. A likely explanation of this latter effect is that when flowering starts early it takes longer to complete because air temperature is colder, while late flowerings occur in a warmer period, which makes them shorter.

### 4.2. Molecular Characterisation

The analysis of the genetic variability and population structure confirmed a high genetic variability and low degree of differentiation (only two groups), with no clear geographic division, apart from the Calabrian varieties. Our results only partially overlap with those of Muzzalupo et al. [31]. This work analyzed a larger set of cultivars (439 Italian varieties) from the same collection, identifying 7 genetic clusters. Main and minor olive varieties were also evaluated by Marra et al. [63] from three distinct regions (Calabria, Sicily, and Campania), indicating more shared allele profiles, especially between Campanian and Calabrian olive germplasm. Discrepancies in olive germplasm genetic analysis results are often due to methodological approaches, such as different sets of SSR markers, no reference authenticated cultivars analyzed, and different instruments used, especially genetic analyzers, leading to great confusion in terms of knowledge about the genetic characterization in olive [30]. Our results tend to align more closely with those of Marra et al. [63], also considering the same methodology and the greater number of common markers used. The parentage analysis seems to confirm the genetic analysis of population structure. Except for Sicily region, where a certain number of geographic barriers can be found justifying cross-pollination among local varieties, in the other cases, the different geographic origins of the putative parentals clearly indicate that gene flow occurred, reducing the genetic differentiation. The “movement” of the olive varieties throughout the Italian peninsula is further confirmed by the presence of several cases of synonymy (seven groups, Table 4) found amongst plants cultivated in different regions.

Some of the synonymies recorded in this work have previously been partially described [31,53], although the following 10 groups of synonymies are being reported for the first time: (1) Carpinetana/Olivella appuntita; (2) Passulunara/Zarbo; (3) Ritonnella/Rotondella lucana; (4) Nasitana a frutto grosso/San Benedetto; (5) Perciasacchi/Ravece; (6) Ogliastro grande/Racioppa campana; (7) Giarfara/Nebba; (8)Carpellese/Frantoio; (9) Dolce di Cerchiara as a synonym of Mafra and Spezzanese; and (10) Arnasca as a synonym of Pesciatino and Ruveia.

**Figure 9:**
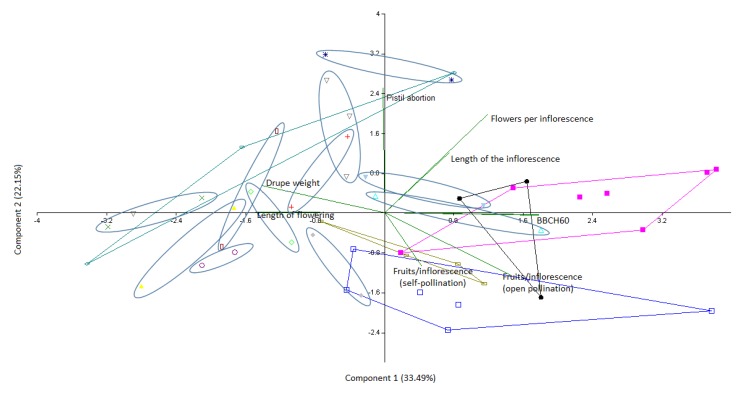
Principal component analysis of the normally distributed physiological parameters obtained from the molecularly revealed cases of synonymy.

Eventually, the normally distributed physiological variables of the varieties found to be synonyms were subjected to a principal component analysis (Figure 9) to evaluate the correspondence with the molecular analyses. Generally, the close clustering of the synonymy groups confirmed microsatellite markers as a reliable system for the discrimination of the olive varieties, whereas the substantial differences found amongst some of the putative synonymous varieties could be due to the comparison between local ecotypes that may have developed phenological, physiological, and structural adaptations to their original environmental conditions.

## 5. Conclusions

This work provides an important contribution to the characterization of the Italian olive biodiversity through the study, at both molecular and physiological levels, of 120 varieties that have traditionally been marginalized. The bio-agronomic characterization provides a basic understanding of the varieties of olive, which is indispensable for not only proper classification, but also for the design and development of future studies that, given the incredibly rich germplasm, are definitely needed in order to increase knowledge about this valuable species. For instance, the identification of a self-fertile cultivar represents a notable datum within what is generally considered a self-sterile species. Furthermore, a greater knowledge of the floral and fruiting biology might allow for the rediscovery and revaluation of many of these varieties, for the purpose of eventually reintroducing them into their areas of origin (or taken beyond to other areas), or for utilization in future breeding programs. In the same regard, the proper varietal identification using molecular markers is an extremely important requirement for the use and marketing of propagation material of agricultural plants. Finally, the observations, based on a large number of varieties and repeated over an eight-year period, aside from providing interesting information on individual cultivars, have allowed us to draw up general rules on the floral biology of the olive tree.

## Figures and Tables

**Table 1:** Characteristic of the examined parameters for the 120 olive varieties with the indication of their regions of origin.

									Fruit Set (%)						
			Flowering			Length of the Inflorescence	Flowers per Inflorescence	Pistil Abortion	Free Pollination (A)		Self-Pollination (B)		Self-Pollination Index	Fruit Drop	Drupe Weight
	Cultivar	Region	Amount	Timing	Length	(mm)	(n)	(%)	Fruits/ Inflorescence	Fruits/ Perfect Flowers	Fruits/ Inflorescence	Fruits/ Perfect Flowers	(B/A)	(%)	(g)
1	Abunara	Sicilia	high	medium	long	26.19	16.31	15.6	28.48	2.07	7.88	0.57	0.28	44.47	6.26
2	Agristigna	Calabria	high	medium	long	23.92	17.46	55.75	16.92	2.19	1.72	0.22	0.1	55.73	6.04
3	Aitana	Sicilia	high	medium	long	30.19	17.05	21.55	21.26	1.59	0.28	0.02	0.01	63.48	3.30
4	Arnasca	Liguria	high	medium	medium	22.01	19.76	12.62	103.34	5.99	4.04	0.23	0.04	27.17	2.57
5	Ascolana dura	Marche	high	late	medium	28.71	14.72	68.7	22.77	4.94	0	0	0	48.42	4.44
6	Aurina	Molise	medium	late	short	26.86	21.28	55.76	80.31	8.53	0.11	0.01	0	36.85	1.37
7	Biancolilla	Sicilia	high	medium	medium	24.05	13.12	29.73	43.65	4.73	5.61	0.61	0.13	83.69	4.16
8	Borgiona	Umbria	medium	medium	medium	37.40	15.14	35.66	30.33	3.11	4.48	0.46	0.15	38.42	3.67
9	Bottone di gallo	Sicilia	high	medium	medium	30.86	20.11	66.19	43.02	6.33	0.54	0.08	0.01	25.27	2.00
10	Cacaredda	Sicilia	medium	early	long	27.28	14.09	11.57	35.83	2.87	4.74	0.38	0.13	34.68	4.23
11	Caizzana	Campania	high	medium	medium	31.09	16.65	20.52	34.97	2.64	0	0	0	28.13	2.24
12	Cammorotana	Campania	medium	medium	medium	22.85	13.37	22.74	30.09	2.91	0.86	0.08	0.03	45.64	4.70
13	Capolga	Marche	high	medium	medium	20.96	14.91	47.51	27.59	3.53	0.21	0.03	0.01	28.74	4.70
14	Caprina di Casalanguida	Abruzzo	medium	early	medium	22.08	12.26	58.08	38.19	7.43	0.1	0.02	0	0.00	2.78
15	Caprina Vastese	Abruzzo	medium	late	short	31.46	18.64	27.74	90.04	6.68	0.36	0.03	0	0.00	2.11
16	Carbonchia	Abruzzo	medium	medium	medium	24.17	12.25	29.62	23.32	2.71	0.1	0.01	0	32.82	1.91
17	Carpellese	Campania	medium	medium	medium	34.89	15.68	7.08	72.64	4.99	19.69	1.35	0.27	27.09	2.44
18	Carpinetana	Abruzzo	high	medium	short	29.49	20.09	39.49	75.7	6.23	0.74	0.06	0.01	23.69	3.47
19	Castricianella rapparina	Sicilia	high	medium	medium	29.49	21.32	50.05	28.18	2.65	0.67	0.06	0.02	23.56	2.05
20	Cavalieri	Sicilia	high	medium	medium	27.24	18.04	41.85	32.18	3.07	0.26	0.03	0.01	29.88	2.90
21	Cellacchia	Lazio	high	late	medium	26.06	20.68	69.06	19.69	3.08	7.29	1.14	0.37	11.44	2.47
22	Cellina di Rotello	Molise	medium	medium	medium	37.64	18.49	12.94	69.58	4.32	30.65	1.9	0.44	32.10	3.07
23	Cicinella	Campania	high	medium	medium	24.62	16.46	34.78	62.53	5.83	4.08	0.38	0.07	30.63	2.31
24	Colombina	Emilia	medium	medium	medium	31.17	18.97	7.32	61.48	3.5	2.82	0.16	0.05	20.46	2.44
25	Corneglia	Campania	low	medium	medium	31.15	16.31	17.64	24.38	1.81	2.67	0.2	0.11	9.84	3.39
26	Cornia	Campania	medium	medium	medium	28.01	10.34	26.43	17.76	2.33	2.26	0.3	0.13	11.39	1.83
27	Corniola	Calabria	high	medium	medium	25.20	18.24	52.51	35.6	4.11	0.74	0.09	0.02	26.25	3.24
28	Crognolo	Lazio	medium	medium	medium	32.31	14.22	1.41	50.52	3.6	17.92	1.28	0.35	21.09	2.50
29	Dolce di Andria	Puglia	medium	early	long	35.95	15.2	11.28	38.69	2.87	2.22	0.16	0.06	78.42	4.49
30	Dolce di Cerchiara	Calabria	medium	medium	medium	27.78	15.11	66.25	19.81	3.88	0	0	0	45.25	3.43
31	Dritta di Loreto	Marche	high	medium	medium	23.40	14.75	22.24	77.42	6.75	0.77	0.07	0.01	32.11	1.82
32	Erbano	Sicilia	high	late	medium	25.72	17.66	42.53	42.07	4.14	3.16	0.31	0.08	39.45	1.85
33	Faresana	Basilicata	high	medium	medium	44.13	21.79	53.46	25.37	2.5	0	0	0	36.47	4.56
34	Femminella di Torraca	Campania	medium	medium	medium	30.60	19.72	35.76	43.11	3.4	2.43	0.19	0.06	33.95	1.97
35	Fosco	Lazio	medium	late	short	38.61	24.59	35.67	50.24	3.18	6.93	0.44	0.14	29.49	2.25
36	Gentile dell’Aquila	Abruzzo	medium	medium	long	24.56	16.35	57.27	35.41	5.07	2.14	0.31	0.06	44.59	4.50
37	Ghiannara	Basilicata	medium	medium	medium	28.34	12.84	18.22	54.51	5.19	6.99	0.67	0.13	35.56	2.34
38	Giarfara	Sicilia	high	medium	long	30.07	14.04	10.86	51.71	4.13	0.19	0.02	0	55.44	5.70
39	Giusta	Basilicata	medium	early	medium	30.04	14.34	15.2	46.8	3.85	0.3	0.02	0.01	20.64	3.69
40	Gnagnaro	Molise	medium	late	short	32.13	19.47	13.52	87.82	5.22	10.11	0.6	0.12	18.76	0.79
41	Grappolo	Toscana	medium	late	medium	29.45	17.73	33.8	115.48	9.84	0.3	0.03	0	31.07	2.53
42	Grossa di Venafro	Molise	medium	late	medium	36.88	28.78	35.35	66.41	3.57	12.5	0.67	0.19	26.91	2.49
43	Grossale	Campania	medium	medium	medium	28.74	18.22	45.32	26.99	2.71	1.53	0.15	0.06	22.58	3.13
44	I/77	Umbria	high	medium	medium	36.15	20.89	81.76	35.25	9.25	0	0	0	46.60	3.79
45	Lumiaru	Sicilia	medium	medium	medium	30.74	18.22	32.75	17.95	1.47	3.24	0.26	0.18	34.20	5.20
46	Mafra	Calabria	high	medium	medium	37.80	25.38	79.23	25.81	4.9	0.51	0.1	0.02	42.58	3.31
47	Monaca	Sicilia	high	medium	medium	29.51	25.08	73.33	15.05	2.25	0.21	0.03	0.01	40.80	4.29
48	Morchiaio	Toscana	high	medium	medium	27.22	18.14	24.35	31.52	2.3	0.38	0.03	0.01	21.79	4.27
49	Morcone	Toscana	high	medium	medium	27.50	21.25	25.47	39.69	2.51	3.78	0.24	0.1	11.26	1.64
50	Morellona di Grecia	Puglia	medium	late	short	30.96	18.8	29.04	88.26	6.62	0.18	0.01	0	32.48	3.13
51	Nasitana a frutto grosso	Sicilia	high	medium	medium	28.38	15.99	21.13	14.75	1.17	0.8	0.06	0.05	22.74	4.20
52	Nebba	Sicilia	high	medium	medium	31.22	15.79	26.1	23.63	2.02	1.43	0.12	0.06	62.03	5.03
53	Nebbia	Marche	medium	medium	medium	28.54	16.68	7.15	99.7	6.44	5.18	0.33	0.05	10.14	2.39
54	Nebbio di Pescara	Abruzzo	medium	medium	medium	40.30	20.69	68.95	42.73	6.65	0.18	0.03	0	19.05	2.46
55	Nera di Colletorto	Molise	medium	medium	medium	31.25	13.08	32.93	50.89	5.8	0.05	0.01	0	18.17	3.15
56	Nerba	Sicilia	medium	medium	medium	23.07	14.01	45.45	22.14	2.9	1.79	0.23	0.08	63.64	4.43
57	Ogliara	Campania	high	medium	medium	29.82	16.43	9.13	58.95	3.95	0.07	0	0	37.22	2.44
58	Ogliastro grande	Campania	high	late	medium	28.01	14.17	52.41	24.62	3.65	0.33	0.05	0.01	33.80	4.71
59	Oliva grossa	Emilia	high	medium	medium	26.96	18.46	90.75	2.27	1.33	0	0	0	45.53	6.97
60	Olivastro di Bucchianico	Abruzzo	medium	medium	medium	35.49	18.63	65.79	19.66	3.08	7.8	1.22	0.4	30.62	2.46
61	Olivastro frentano	Abruzzo	high	late	short	34.00	18.48	82.84	50.45	15.91	0.28	0.09	0.01	18.90	1.86
62	Olivella appuntita	Campania	high	late	short	24.83	18.03	38.17	69.14	6.2	1.93	0.17	0.03	33.10	3.19
63	Olivella di Cerchiara	Calabria	high	medium	medium	29.26	20.71	84.07	25.28	7.66	0.43	0.13	0.02	31.28	3.45
64	Olivo da mensa	Basilicata	high	medium	medium	31.34	17.58	67.85	13.86	2.45	0.09	0.02	0.01	34.73	4.60
65	Olivo da olio	Campania	high	medium	medium	28.58	14.91	12.74	58.94	4.53	0.23	0.02	0	22.18	2.42
66	Olivo da salare	Campania	medium	medium	long	26.94	13.12	26.37	30.28	3.13	0.93	0.1	0.03	63.70	3.17
67	Olivo di Casavecchia	Toscana	medium	medium	medium	30.48	13.8	16.85	22.08	1.92	0.4	0.03	0.02	22.90	3.06
68	Olivo di Castiglione	Sicilia	high	medium	medium	20.00	10.56	9.7	18.21	1.91	0.82	0.09	0.05	59.00	4.51
69	Olivone di Viterbo	Lazio	high	medium	medium	40.01	28.16	88.93	37.18	11.93	7.76	2.49	0.21	28.00	3.23
70	Ornellaia	Toscana	high	medium	medium	36.46	13.77	30.72	41.92	4.39	4.7	0.49	0.11	29.81	4.16
71	Ortice	Campania	medium	medium	medium	34.54	22.84	58.17	45.27	4.74	0.15	0.02	0	25.89	2.54
72	Paesana bianca	Molise	medium	medium	medium	35.29	16.92	10.69	35.38	2.34	9.79	0.65	0.28	51.40	2.45
73	Paesana nera	Molise	high	late	short	34.26	23.18	29.86	69.38	4.27	2.88	0.18	0.04	43.37	2.49
74	Palmarola	Basilicata	medium	late	medium	26.37	12.7	2.6	62.67	5.07	3.54	0.29	0.06	61.79	2.35
75	Passulunara	Sicilia	medium	medium	medium	31.98	11.87	8.55	43.4	4	14.1	1.3	0.32	50.05	4.87
76	Pennulara	Calabria	medium	medium	medium	24.29	10.64	13.22	21.62	2.34	21.94	2.38	1.01	62.05	4.61
77	Perciasacchi	Campania	high	medium	medium	27.02	20.97	76.06	29.69	5.91	0.9	0.18	0.03	40.66	3.04
78	Pesciatino	Toscana	medium	medium	medium	25.08	22.45	76.06	89.58	16.67	1.09	0.2	0.01	17.00	2.26
79	Piangente	Toscana	high	medium	long	32.65	17.74	4.85	67.01	3.97	0.77	0.05	0.01	28.74	2.58
80	Pizzutella	Sicilia	high	medium	medium	21.19	24.41	68.1	8.1	1.04	0.11	0.01	0.01	19.74	3.56
81	Posola	Abruzzo	medium	late	medium	22.53	15.51	35.52	27.73	2.77	0.22	0.02	0.01	37.36	3.81
82	Posolella	Abruzzo	medium	medium	medium	35.28	18.79	36.4	48.01	4.02	0.7	0.06	0.01	21.02	1.91
83	Procanica	Lazio	medium	medium	short	35.06	15.87	1.07	90.44	5.76	2.49	0.16	0.03	34.41	2.54
84	Provenzale	Campania	high	early	long	26.02	13.59	49	22.31	3.22	4.68	0.68	0.21	9.01	3.09
85	Puntella	Abruzzo	medium	late	medium	31.34	21	71.55	49.57	8.3	3.91	0.65	0.08	23.07	2.05
86	Racioppa campana	Campania	high	late	medium	31.06	21.38	13.57	47.46	2.57	0.71	0.04	0.01	26.02	3.50
87	Racioppa	Basilicata	medium	medium	medium	24.82	19.58	31.21	107.62	7.99	0.55	0.04	0.01	24.10	2.41
88	Rajo	Umbria	high	medium	long	37.06	15.43	28.32	62.91	5.69	1.49	0.14	0.02	13.92	2.48
89	Ravece	Campania	high	medium	medium	25.68	14.76	51.74	36.88	5.18	1.19	0.17	0.03	36.15	2.99
90	Remugnana	Molise	medium	medium	medium	32.59	18.28	2.26	81.07	4.54	0.96	0.05	0.01	26.87	1.96
91	Resciola di Venafro	Molise	medium	late	short	35.36	19.99	44.47	49.36	4.45	6.84	0.62	0.14	20.29	2.55
92	Riminino	Lazio	high	early	medium	46.65	28.59	76.56	39.32	5.87	2.82	0.42	0.07	53.71	3.82
93	Ritonnella	Campania	high	medium	long	27.32	21.19	69.95	12.93	2.03	4.08	0.64	0.32	58.93	3.54
94	Rizzitella	Campania	medium	medium	short	24.08	17.66	57.25	26.32	3.49	1.96	0.26	0.07	45.36	2.15
95	Rosciola coltodino	Lazio	high	early	medium	36.49	24.04	61.69	82.01	8.9	1.13	0.12	0.01	28.01	1.85
96	Rosciola di Rotello	Molise	medium	medium	medium	31.59	13.55	9.23	59.03	4.8	5.08	0.41	0.09	30.75	2.37
97	Rossina	Emilia	high	medium	medium	21.23	14.18	30.1	62.99	6.36	3.34	0.34	0.05	38.93	1.52
98	Rotondella campana	Campania	high	medium	long	27.50	15.52	45.38	49.71	5.87	2.88	0.34	0.06	52.27	3.31
99	Rotondella lucana	Basilicata	high	medium	medium	28.20	12.25	30.41	23.47	2.75	6.63	0.78	0.28	12.27	2.91
100	Rustica	Abruzzo	high	medium	long	27.71	15.41	66.52	27.65	5.36	0.46	0.09	0.02	29.46	3.88
101	Ruveia	Campania	high	medium	short	21.21	19.11	55.66	52.89	6.24	0	0	0	16.76	2.23
102	Saligna	Molise	high	late	medium	30.73	18.92	46.42	62.07	6.12	17.63	1.74	0.28	25.70	2.24
103	Sammartinara	Sicilia	medium	medium	medium	30.23	14.16	34.31	56.66	6.09	0.34	0.04	0.01	31.19	3.76
104	Sammartinenga	Basilicata	high	early	long	29.50	22.99	80.84	20.04	4.55	0	0	0	38.36	1.81
105	San Benedetto	Puglia	high	early	long	28.64	15.65	10.18	28.69	2.04	0.09	0.01	0	23.91	3.93
106	Sanginara	Campania	high	medium	medium	29.09	20.18	43.82	45.27	3.99	0.95	0.08	0.02	21.52	1.96
107	Santa Maria	Campania	medium	late	short	37.47	15.58	39.11	73.69	7.77	2.81	0.3	0.04	17.85	1.91
108	Scarpetta	Basilicata	medium	late	medium	29.29	15.98	27.91	86.06	7.47	0.43	0.04	0.01	21.75	2.11
109	Sessana	Campania	high	late	medium	26.51	17.71	50.02	42.13	4.76	7.36	0.83	0.17	23.54	1.98
110	Sivigliana da olio	Sardegna	medium	medium	medium	33.20	26.21	89.24	35.77	12.68	0	0	0	35.59	1.73
111	Spagnola di Missano	Liguria	high	medium	medium	32.36	21.51	74.48	52.66	9.59	7.05	1.28	0.13	39.64	3.61
112	Spezzanese	Calabria	high	early	long	24.86	13.95	19.28	29.48	2.62	14.46	1.28	0.49	30.31	5.70
113	Tenacella	Campania	high	late	short	31.79	18.98	61.68	73.82	10.15	33.76	4.64	0.46	26.57	1.01
114	Tombarello	Calabria	high	medium	medium	27.45	19.34	13.25	37	2.2	0.33	0.02	0.01	22.44	1.79
115	Tonda di Alife	Campania	high	late	medium	31.16	22.13	42.77	57.91	4.57	8	0.63	0.14	31.09	2.70
116	Tonda dolce	Calabria	medium	early	medium	25.84	14.26	25.45	27.74	2.61	0.17	0.02	0.01	43.28	2.89
117	Tunnulidda	Sicilia	medium	early	long	27.18	15.73	14.9	37.97	2.84	5.31	0.4	0.14	37.60	4.13
118	Vigna della corte	Campania	medium	medium	medium	21.25	12.71	30.97	14.32	1.63	1.12	0.13	0.08	0.00	2.72
119	Vocio	Umbria	medium	medium	medium	25.51	15.17	36.32	23.57	2.44	5.17	0.54	0.22	20.23	4.09
120	Zarbo	Sicilia	low	medium	medium	29.59	13.41	29.4	64.8	6.85	1.51	0.16	0.02	33.81	5.66

**Table 2:** Parameters of genetic diversity for each SSR marker.

Locus	Na	Ho	He	HW	F (null)	PIC	NE-I	NE-SI
dca3	11	0.916	0.847	*	−0.0421	0.824	0.044	0.339
dca5	15	0.831	0.696	***	−0.1179	0.673	0.114	0.432
dca8	18	0.975	0.845	**	−0.0760	0.822	0.044	0.340
dca16	23	0.689	0.858	**	0.1122	0.843	0.033	0.331
dca18	12	0.957	0.852	NS	−0.0607	0.831	0.041	0.336
gapu71b	8	0.881	0.784	***	−0.0626	0.745	0.084	0.381
emo090	8	0.786	0.713	NS	−0.0621	0.672	0.122	0.426
dca9	22	0.924	0.898	NS	−0.0182	0.884	0.021	0.308
udo15	8	0.75	0.858	ND	ND	0.78	0.063	0.363
udo12	3	0.733	0.66	NS	−0.0842	0.565	0.204	0.482
dca11	10	0.379	0.771	***	0.3390	0.735	0.085	0.389
olest23	3	0.4	0.511	ND	ND	0.41	0.341	0.605
Mean	11.75	0.77	0.77			0.73		

Allele number (Na); observed heterozygosis (Ho); expected heterozygosis (He); statistical significance of HW test *** *P* < 0.01, * *P* < 0.05; probability of null alleles (F); polymorphism information content (PIC); the non-exclusion probability between two unrelated individuals (NE-I); and two hypothetical full siblings (NE-SI).

**Table 3:** Rare alleles detected for each microsatellite locus in the 120 olive varieties.

Cultivar	Provenience	Allele Size	Locus
Carbonchia	Abruzzo	159	DCA18
Rustica	Abruzzo	202	DCA9
Sammartinenga	Basilicata	241	DCA3
Sammartinenga	Basilicata	164	DCA16
Scarpetta	Basilicata	184	EMO090
Giusta	Basilicata	196	DCA5
Corniola	Calabria	135	DCA8
Dolce di Cerchiara	Calabria	162	DCA16
Santa Maria	Campania	255	DCA3
Corneglia	Campania	204	DCA5
Olivone di Viterbo	Lazio	136	DCA16
Gnagnaro	Molise	134	DCA16
Rosciola di Rotello	Molise	196	EMO090
Gnagnaro	Molise	130	DCA11
Biancolilla	Sicilia	210	DCA9
Bottone di Gallo	Sicilia	129	DCA8
Castricianella Rapparina	Sicilia	161	DCA8
Castricianella Rapparina	Sicilia	192	EMO090
Erbano	Sicilia	166	DCA9
Rajo	Umbria	188	DCA9

**Table 4:** List of the 17 synonymy groups found amongst the 120 olive varieties, their geographic origin, genetic similarity (Dice’s index), and the corresponding group found with the population structure analysis.

Cultivar	Geographic Provenience	Dice Similarity Index	Structure Group
Carpinetana	Abruzzo	0.87	admixture
Olivella Appuntita	Campania		admixture
Arnasca	Liguria	0.84	Red
Pesciatino	Toscana	1	Red
Ruveia	Campania		Red
Ogliara	Campania	0.87; 0.77; 0.77	Red
Olivo da olio	Campania	0.9; 0.9	Red
Gentile Nera di Colletorto	Molise	0.87	Red
Remugnana	Molise		Red
Faresana	Basilicata	0.86	admixture
Olivo da Mensa	Basilicata		admixture
Ritonnella	Campania	0.9	admixture
Rotondella lucana	Basilicata		admixture
Dolce di Cerchiara	Calabria	0.87; 0.81	Red
Mafra	Calabria	0.87	Red
Spezzanese	Calabria		Red
Nasitana Frutto Grosso	Sicilia	0.94	Red
San Benedetto	Puglia		Red
Cacaredda	Sicilia	0.94	Red
Tunnulidda	Sicilia		Red
Nerba	Sicilia	0.86	Red
Olivo di Castiglione	Sicilia		Red
Perciasacchi	Campania	1	admixture
Ravece	Campania		admixture
Grossale	Campania	0.84	Red
Provenzale	Campania		Red
Ogliastro grande	Campania	0.93	Red
Racioppa Campana	Campania		Red
Giarfara	Sicilia	0.96	Green
Nebba	Sicilia		Green
Carpellese	Campania	0.77; 0.84;0.84; 0.81;0.71	Green
Crognolo	Lazio	0.94; 0.93; 0.92; 0.8	Green
Ghiannara	Basilicata	1; 1; 0.87	Green
Paesana Bianca	Molise	1; 0.87	Green
Procanica	Lazio	0.85	Green
Rosciola di Rotello	Molise		Green
Caprina vastese	Abruzzo	0.83; 0.83; 0.83; 0.8; 0.76; 0.77	Green
Cicinella	Campania	1; 1; 0.9; 0.93; 0.87	Green
Sanginara	Campania	1; 0.9; 0.93; 0.87	Green
Tonda di Alife	Campania	0.9; 0.93; 0.87	Green
Fosco	Lazio	0.9; 0.9	Green
Grossa di Venafro	Molise	0.94	Green
Paesana nera	Molise		Green
Olivone di Viterbo	Lazio	0.96	admixture
Riminino	Lazio		admixture
Passulunara	Sicilia	0.83	Red
Zarbo	Sicilia		Red

**Table 5:** Parentage analysis results. The table shows the olive cultivars identified as putative parents of the offspring cultivars, and the corresponding regions of origin.

Offspring ID	Region	First Candidate ID	Region	Second Candidate ID	Region
Abunara	Sicily	Giarfara	Sicily	Scarpetta	Basilicata
Aitana	Sicily	Giarfara	Sicily	Scarpetta	Basilicata
Capolga	Marche	Morellona di Grecia	Puglia	Rossina	E. Romagna
Colombina	E. Romagna	Carpellese	Campania	Palmarola	Basilicata
Gentile dell’Aquila	Abruzzo	Fosco	Lazio	Posola	Abruzzo
Grappolo	Tuscany	Paesana Bianca	Molise	Piangente	Tuscany
I/77	Umbria	Cicinella	Campania	Olivo di Castiglione	Sicily
Lumiaru	Sicily	Caiazzana	Campania	Rustica	Abruzzo
Oliva grossa	E. Romagna	Spagnola di Missano	Liguria	Zarbo	Sicily
Olivo da salare	Campania	Arnasca	Liguria	Bottone di gallo	Sicily
Palmarola	Basilicata	Colombina	E. Romagna	Paesana bianca	Molise
Rizzitella	Campania	Bottone di gall	Sicily	Racioppa lucana	Basilicata
Rosciola coltodino	Lazio	Carpinetana	Abruzzo	Racioppa lucana	Basilicata
Rossina	E. Romagna	Giarfara	Sicily	Paesana near	Molise
Sammartinara	Sicily	Nebba	Sicily	Scarpetta	Basilicata
Santa Maria	Campania	Aurina	Molise	Rossina	E. Romagna
Sessana	Campania	Procanica	Lazio	Vigna della Corte	Campania
Spagnola di Missano	Liguria	Carpellese	Campania	Oliva grossa	E. Romagna
Vigna della corte	Campania	Monaca	Sicily	Rotondella lucana	Basilicata
Vocio	Umbria	Carpinetana	Abruzzo	Olivo da salare	Campania
Zarbo	Sicily	Giarfara	Sicily	Scarpetta	Basilicata
	One of the parental genotypes and the offspring share the same region of origin.				
	Neighboring regions of origin for the two parental genotypes.				
	No type of geographical relationship.				

## Supplementary Materials

The following are available online at https://www.mdpi.com/2079-7737/8/3/62/s1, Table S1: Models for predicting flowering date which were tested, Table S2: Results of the cross validation test for all the models under study, Table S3: Specific cumulated heat requirement for each cultivar under study, calculated by fitting SW1 model (Table S1) to data.
