# Supplementary material for: Uncovering Olive Biodiversity through Analysis of Floral and Fruiting Biology and Assessment of Genetic Diversity of 120 Italian Cultivars with Minor or Marginal Diffusion
Source: Biology (Basel). 2019 Aug 28;8(3):62. doi: 10.3390/biology8030062 (PMC6783865; doi:10.3390/biology8030062)
Supplement: Supplementary file 1 [file biology-08-00062-s001.pdf]

SUPPLEMENTARY MATERIALS

Model-based phenology characterization

Model description

The target model was selected among ten candidate models belonging to basically two types: i) Spring Warming models (SW), which accumulates heat units (HU) starting from a fixed date, typically the 1st of January, until flowering occurs once a critical sum is reached, and ii) Chilling and Forcing models (CF), where HU summation is started from dormancy break, which in turn is estimated by calculation of a critical sum of chilling units (CU) starting from the 1st September of the year preceding flowering.

CUs are calculated as days below a given critical temperature or derived from the daily mean temperature input to a beta-like temperature response function, so named because of its similarity to the beta distribution (Yan & Hunt, 1999), whose parameters are three cardinal temperatures, minimum (Tn), optimum (To) and maximum temperature (Tx). The number of parameters can be reduced to two if Tn and Tx are symmetric respect to To, so they can be defined through a same distance from To. HUs are calculated as growing degree days (GDD, °C d), with T base (Tb) optimized against data for each cultivar. By taking a same Tb value for all cultivars, obtained by averaging all optimized values, a one-parameter GDD model can be built, which is the simplest model that can be build. In the same way as CUs, HUs can also be derived from beta-like functions from mean daily temperature.

To further reduce the total number of parameters, a simplifying assumption was also considered: cardinal temperatures of beta-like temperature response function for CUs and HUs calculation were considered constant for all varieties, while only the critical sums varied. The temperature constants were derived after averaging the optimized temperatures sourced from models using the 3-parameters version of the function. By this way, a 4-parameter SW models (3 parameters for the beta-like function plus the critical sum) reduces to one parameter. The objective of this approach is to reduce overfitting effects.

A number of SW and CF models were built as combinations of such criteria, which are listed in Table 1 with a short description. All these models are daily step, and use mean daily temperature as daily input to CU and HU calculation.

The model developed by De Melo-Abreu et al (2004) was included and coded as CF5. Unlike the other models, this one uses a hourly time step for CU calculation and a daily time step for HU. CU are calculated with a piecewise defined function, which allows for negative CU when a maximum temperature is exceeded.

As a reference, a null model was defined, consisting in just the historical average of the flowering dates for each cultivar.

Model cross-validation

The models were cross-validated against the Mirto dataset by means of a genetic algorithm, using a leave-one-out cross-validation (LOOCV) procedure. As a reference, a null model was defined, consisting in just the historical average of the flowering dates for each cultivar.

Since eight observational dates were available for almost all cultivars, the procedure yielded eight flowering estimates and as many predicted residuals (PR, i.e. difference between predicted and observed flowering date) for each cultivar.

Final model selection was based on the cross-cultivars average PR, and Akaike Information Criterion (AIC). PR index gives an immediate grasp of the prediction accuracy of a model. However, in a cross validation procedure, models are iteratively trained on different datasets; ideally, since the underlying process described by a model is always the same independently from the data it applies to, a given model should converge to equal or similar parameters in all datasets. In fact, especially on more complex models, parameters can assume quite different values when trained to different datasets, and be capable of giving good validation fit all the same. AIC is a useful indicator to quantify model consistency through the cross-validation cycles, weighing the effect of complexity, so that to prevent or attenuate overfitting effects.
Once a best model was individuated, it was finally optimized on the whole dataset.

Table S1. Models for predicting flowering date which were tested. (CU = chilling units; HU = heat units; T = mean daily temperature; Tb = base temperature, p. = parameters; DOY = day of the year; GDD = growing degree days).

model	CU calculation	HU calculation	N of p.
SW1		GDD with Tb=average of optimized values° C for all cultivars	1
SW2		GDD with optimized Tb	2
SW3		3-p. beta-like function	4
SW4		2-p. beta-like function	3
SW5		beta-like function with 3 constant p. for all cultivars	1
CF1	number of days below an optimized T	GDD with optimized Tb	4
CF2	3-p. beta-like function	3-p. beta-like function	8
CF3	2-p. beta-like function	2-p. beta-like function	6
CF4	beta-like function with 3 constant p. for all cultivars	beta-like function with3 constant p. for all cultivars	2
CF5	3-p. piece-wise function, hourly time step considers negative chilling units	GDD with optimized Tb	5
Null	Historical DOY of flowering date as estimator		0

Cross-Validation results

In terms of accuracy (RMSE) the best performing model was CF2, with 1.33 days. However, when prediction accuracy was balanced with complexity, SW1 model, corresponding to the traditional GDD model with an optimized average Tbase of 2.25 °C, resulted the best one, with the minimum AIC index of 10.13. This means that SW1, respect to SW2 which was derived from, gives a more consistent parameterization across cross validation iterations, which was a rather expected result as it has one parameter only. Despite this extreme simplicity, this model generates a reasonably good validation fit of 2.54.
SW1 was therefore selected as model to characterize cultivar earliness, with the advantage of doing that with just one variable, i.e. the GDD sum.

Table S2. Results of the cross validation test for all the models under study (PR = predicted residuals, i.e. mean difference between predicted and observed flowering date; AIC = Akaike Information Criterion indicator). The best obtained indices are evidenced in bold characters.

model	PR	AIC
SW1	2.54	**10.13**
SW2	2.43	12.36
SW3	2.35	22.08
SW4	2.45	19.00
SW5	2.57	14.00
CF1	2.13	19.68
CF2	**1.33**	21.60
CF3	2.12	24.55
CF4	3.31	17.74
CF5	1.38	16.21
null	5.40	21.72

Table S3. Specific cumulated heat requirement for each cultivar under study, calculated by fitting SW1 model (Table S1) to data. Values are listed in decreasing order.

CULTIVAR	GDD sum
rosciola coltodino	1251.0
tonda dolce	1251.0
giusta	1251.1
spezzanese	1251.1
dolce di andria	1251.2
san benedetto	1251.4
sammartinenga	1256.8
caprina di casalanguida	1259.9
provenzale	1260.5
olivo da salare	1265.8
abunara	1265.9
riminino	1266.0
paesana bianca	1277.1
carpinetana	1277.4
pennulara	1278.8
nasitana frutto grosso	1292.5
olivo di castiglione	1292.6
corniola	1294.9
ghiannara	1294.9
cicinella	1295.0
nera di colletorto	1295.0
rizzitella	1295.1
biancolilla	1295.3
capolga	1295.3
ritonnella	1295.3

rustica	1295.4
vocio	1295.4
caiazzana	1295.5
tombarello	1295.5
faresana	1295.6
nebbia	1295.6
ravece	1295.6
vigna della corte	1295.6
aitana	1295.7
cacaredda	1295.7
dolce di cerchiara	1295.7
nebba	1295.7
rotondella lucana	1295.7
cavalieri	1295.8
cellina di rotello	1295.8
cornia	1295.8
lumiaru	1295.8
agristigna	1295.9
morchiaio	1295.9
remugnana	1295.9
corneglia	1296.0
nerba	1296.0
olivella di cerchiara	1296.0
rotondella campana	1296.0
spagnola di missano	1296.0
tunnulidda	1296.0
castricianella rapparina	1296.1
gentile dell'aquila	1296.1
olivone di viterbo	1296.1
sammartinara	1296.1
procanica	1296.2
cammarotana	1309.2
ogliara	1309.2
bottone di gallo	1309.3
ornellaia	1309.4
rosciola di rotello	1309.5
posolella	1309.6
monaca	1309.8
perciasacchi	1309.8
i 77	1309.9
pesciatino	1309.9
nebbio di pescara	1310.0
puntella	1310.0
scarpetta	1310.1
colombina	1310.2
borgiona	1310.3
pizzutella	1310.3

olivastro di bucchianico	1310.5
sessana	1310.6
mafra	1310.8
oliva grossa	1310.8
crognolo	1310.9
zarbo	1310.9
grossale	1311.0
ruveia	1311.1
olivo da mensa	1311.5
femminella di torraca	1311.6
giarfara	1311.9
rajo	1324.4
passulunara	1325.0
dritta di loreto	1325.5
olivo di casavecchia	1325.7
morcone	1325.8
olivo da olio	1325.8
palmarola	1327.1
sivigliana da olio	1327.2
arnasca	1327.7
ortice	1328.1
tenacella	1329.0
sanginara	1330.4
olivastro frentano	1330.6
paesana nera	1341.1
carbonchia	1342.0
posola	1343.5
olivella appuntita	1343.8
morellona di grecia	1344.0
ogliastro grande	1345.5
rossina	1346.0
tonda di alife	1346.5
resciola di venafro	1346.9
caprina vastese	1347.1
grappolo	1361.5
piangente	1361.9
grossa di venafro	1367.5
cellacchia	1367.6
saligna	1367.6
santa maria	1367.7
ascolana dura	1368.0
erbano	1368.6
racioppa	1368.7
aurina	1380.5
fosco	1380.5
gnagnaro	1380.5
carpellese	1380.8
